# A Case Report: Nager Acrofacial Dysostosis

**Published:** 2012

**Authors:** Shahin Abdollahi Fakhim, Nikzad Shahidi, Mehrnoush Mousaviagdas

**Affiliations:** 1 Department of otorhinolaryngology, Tabriz University of Medical Sciences, Tabriz, Iran

**Keywords:** Anomaly, Craniofacial, Nager syndrome

## Abstract

**Introduction:**

Nager syndrome is a malformation resulting from problems in the development of the first and second branchial arches and limb buds. The cause of the abnormal development of the pharyngeal arches in Nager syndrome is unknown. It is also unclear why affected individuals have bone abnormalities in their arms and legs. Nager syndrome is thought to have an autosomal recessive inheritance pattern when unaffected parents have more than one affected child. The purpose of this report is to present a case of Nager syndrome where the patient exhibited upper limb shortening, an unusual feature that has been reported as coexisting in some individuals with Nager syndrome.

**Case report:**

A 3.5-year-old girl was referred to our Department of Pediatric Otorhinolaryngology due to a cleft palate. Her craniofacial anomalies included malar hypoplasia, severe mandibular hypoplasia with retrognathia, downward slanted palpebral fissures, a high narrow hard palate, absent soft palate, small retroplaced tongue, bilateral external auditory canal atresia, and dysplastic ears. There was no evidence of mental retardation. Based on the craniofacial characteristics and the coexisting upper limb preaxial anomalies, a diagnosis of Nager syndrome was confirmed.

**Conclusion:**

Nager syndrome is a rare disorder resulting from developmental abnormalities of the first and second branchial arches. It is linked to five other similar syndromes: Miller syndrome, Treacher-Collins, Pierre-Robin, Genee-Wiedemann, and Franceschetti-Zwahlen-Klein. Multidisciplinary management by a craniofacial team is needed. Early intervention, intensive education, new surgical techniques, and an emphasis on coordinated care have improved the quality of life in this patient with Nager syndrome.

## Introduction

Nager syndrome is a malformation resulting from problems in the development of the first and second branchial arches and limb buds (1, 2). It was first recognized in a patient and reported by Nager and de Reynier in 1948 (3), who used the term acrofacial dysostosis to distinguish the condition from mandibulofacial dysostosis (4). The cause of the abnormal development of the pharyngeal arches in Nager syndrome is unknown. The first arches produce the nerves and muscles for chewing, the lower jaw, two of the three bones in the middle ear, and a small part of the ears. The second arches produce the nerves and muscles of facial expression and one bone in the middle ear (5). Most cases of Nager syndrome are sporadic (6). When the disorder is familial it can have either an autosomal dominant (7-12) or an autosomal recessive pattern of inheritance (13-17). Nager syndrome is thought to have an autosomal recessive inheritance pattern when unaffected parents have more than one affected child.

Major symptoms of Nager syndrome may include underdevelopment of the cheek and jaw area of the face, down-sloping of the opening of the eyes, lateral coloboma of the lower eyelids, a reduced number of eyelashes or absence of the lower eyelashes, kidney and/or stomach reflux, a smaller than normal jaw, lack of development of the internal and external ear with related hearing problems, cleft palate, hammer toes, shortened forearms due to the partial or complete absence of the radius bone, difficulty fully extending the elbows, bone abnormalities in the legs and feet, and shortened soft palate (2,3,6,18-21). Although speech development may be delayed due to hearing impairment, Nager syndrome does not affect a child’s intelligence. Diagnosis is made based on the clinical features, patient history, and genetic testing of the child and parents. Several surgeries may be necessary depending on the severity of the condition. Some surgeries which may be needed include a tracheostomy to help with breathing, a gastrostomy tube to assure proper nutrition, and craniofacial surgery to the jaw and ears. 

The purpose of this report is to present a case of Nager syndrome where the patient exhibited upper limb shortening, a feature that has been reported as coexisting in some individuals with the syndrome.

## Case Report

A 3.5-year-old girl was referred to our Department of Pediatric Otorhinolaryngology because of her cleft palate. A general assessment showed evidence of malformations in other areas. The mother’s history regarding alcohol, smoking, and drug abuse was negative and there was no family history of congenital abnormalities or consanguinity. The mother’s history of fetal infections and drug use in pregnancy was also negative. The child was visited periodically from birth by pediatricians because of her problems. The child had feeding difficulties owing to malformations of the orofacial region, most important of which was a severe mandibular retrusion, cleft palate, and tongue-tie. 

**Fig 1:**
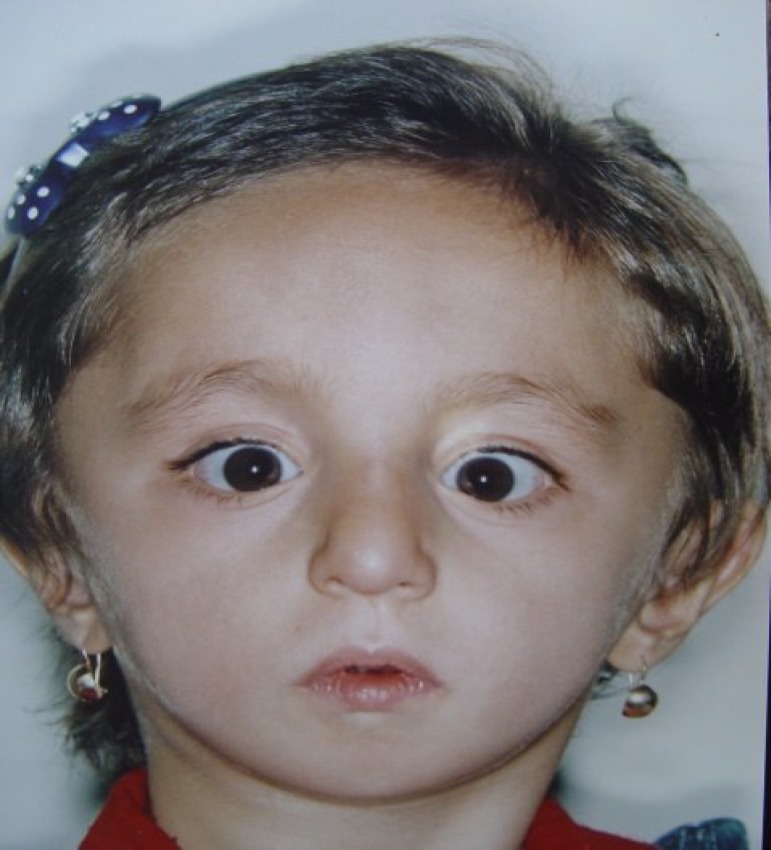
Malar hypoplasia, severe mandibular hypoplasia with retrognathia

**Fig 2:**
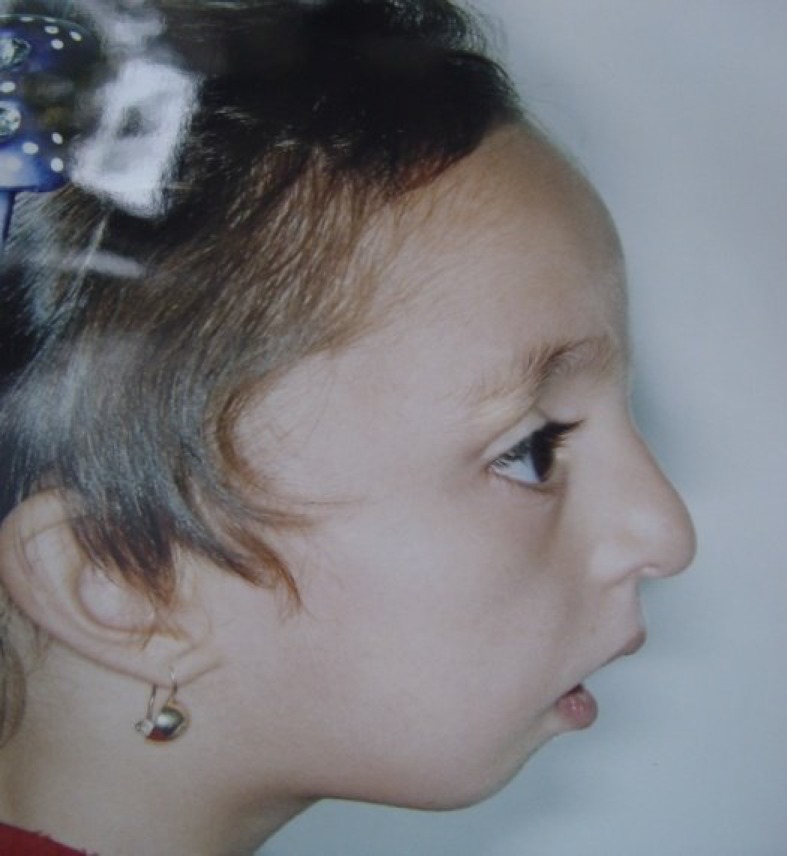
Downward slanted palpebral fissures

**Fig 3:**
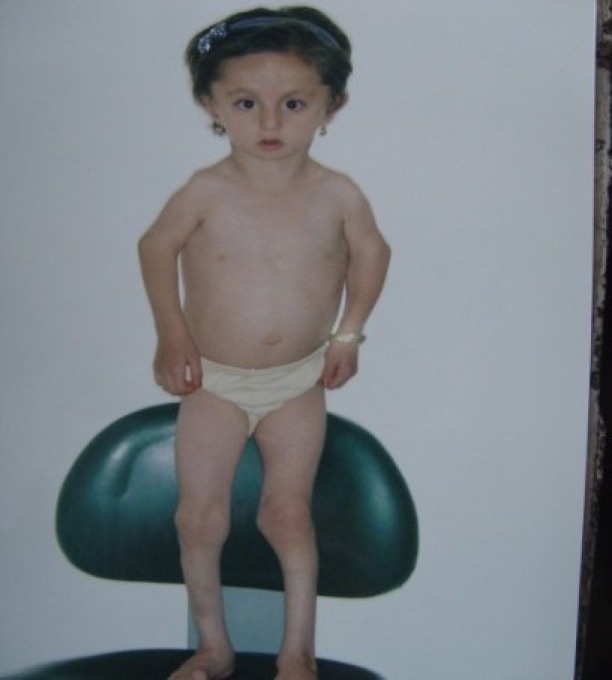
Genu varum and bilateral club feet

Clinical examination at the age of 3.5 years showed a child with growth retardation evidenced by a height of 77 cm (below 3^rd^ percentile), weight of 8800 g (below the 3rd percentile), and a head circumference of 47 cm. Craniofacial anomalies present included malar hypoplasia, severe mandibular hypoplasia with retrognathia (see Fig. 1), downward slanted palpebral fissures (see Fig. 2), a high narrow hard palate, absent soft palate, small retroplaced tongue, bilateral external auditory canal atresia, and dysplastic ears. There was no evidence of mental retardation. The arms were short and elbow articulation had motion limitations in extension and flexion. In the lower limbs there were genu varum and bilateral club feet (Fig. 3). The patient also exhibited characteristic facies owing to hypoplastic zygomatic regions and mandibular retrusion. There was no restriction of jaw movement. 

**Fig 4:**
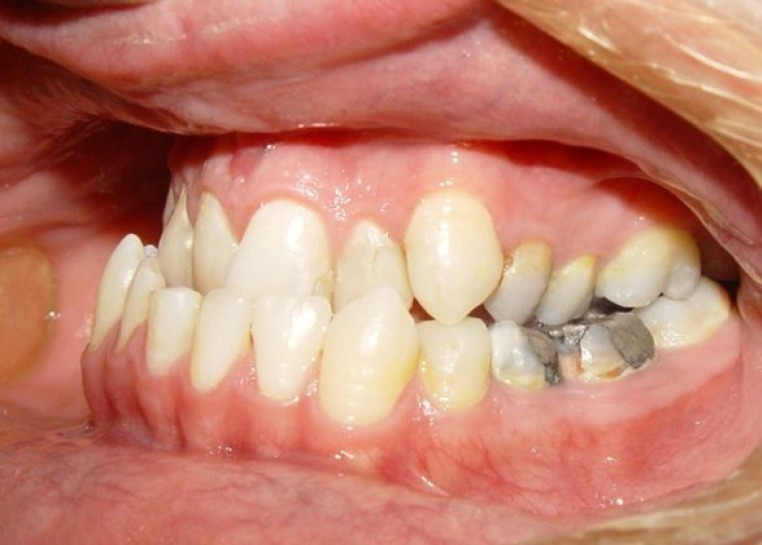
Abnormal overbite with overlap of the upper teeth

At the time of examination she had speech and feeding difficulties. The feeding difficulties were attributable to velopharyngeal incompetence, cleft palate, and ankyloglosia. Ocular findings included anisopthalmia, anisocoria, cloboma and downslanting palpebral fissures due to the zygomatic hypoplasia. In addition, dysplastic low-set ears were observed. Hearing level was 50 dB using an audiogram. Dental and dentoskeletal relationships were considered within the normal range. However, the lower incisors had a lingual position and they had an abnormal overbite with overlap of the upper teeth (see Fig. 4). Radiographic hand and wrist examination provided an estimated bone age of 1.5 years.

The thyroid and genitourinary tract were normal in function according to sonography. There was evidence of neither respiratory disease or of hormonal disturbance (growth hormone = 1.3). Mild cardiovascular abnormalities were seen in an echocardiogram including a small Patent Dactus Arteriosum (PDA) and mild left ventricular dilation. Blood amino acid chromatography was negative. Based on the patient’s craniofacial characteristics and the coexisting upper limb preaxial anomalies, a diagnosis of Nager syndrome was confirmed.

Early intervention was not problematic because of the parents’ previous education in other medical centers. The patient was 3.5 years old and bought to the hospital by her parents because of a cleft palate. Surgical intervention was undertaken to improve this defect.

## Discussion

Nager syndrome is a rare disorder resulting from developmental abnormalities of the first and second branchial arches (2) and is linked to five other similar syndromes: Miller syndrome, Treacher-Collins, Pierre-Robin, Genee-Wiedemann, and Franceschetti-Zwahlen-Klein. Although most cases are sporadic, acrofacial dysostosis has now been reported in siblings and in the children of a consanguineous mating, suggesting autosomal recessive inheritance. It is imperative that acrofacial dysostosis be recognized as an entity distinct from the more common autosomal dominant condition, mandibulofacial dysostosis (Treacher-Collins syndrome) (22). 

The facial features of Nager syndrome grossly resemble those of Treacher-Collins syndrome; however, differentiation between the two can be made. Patients with Treacher-Collins syndrome have more severe forms of hypoplastic zygoma, downward slanting palpebral fissures, lower lid colobomata, and hypoplastic maxillae (23). In general, these particular characteristics are less severe in Nager syndrome. Typical of Nager syndrome is a more extreme degree of mandibular hypoplasia, a greater frequency of palate abnormalities, and limb anomalies (24). Sulik and colleagues (1, 25) have suggested that the pathogenesis of Nager syndrome may be attributed to disturbances in development of the proximal aspects of the maxillary and mandibular prominences of the first branchial arch and the apical ectodermal ridges of the limb buds (13-17).

While the patient had a severe degree of mandibular hypoplasia and limitation of mandibular motion with severe trismus, her limb anomalies were not unusually severe. The craniofacial characteristics and coexisting upper limb preaxial anomalies of Nager syndrome are listed in (Table 1) (4) along with the positive findings in this case.

**Table 1:** Clinical and radiographic features commonly encountered in preaxial acrofacial dysostosis (Nager syndrome)

**Common features **	**Presentcase**
Maxillofacial area	
Zygomatic hypoplasia	+
Downslanting palpeblar fissures	+
Lower eyelid colobomas	+
Mandibular hypoplasia	+
Ear involvement	
External ear malformation	+
Limbs	
Thumb aplasia or hypoplasia	
Radial defects	
Others	
Genitourinary abnormalities	
Reduced stature	

Many of the previous cases of Nager syndrome reported were identified when the children were newborn, therefore their intellectual capability could not be assessed. Of the previously reported cases, 17 (49%) were between the ages of 16 months and 36 months and only two (12%) were reported as mentally retarded (24). Our patient did not have symptoms of mental retardation. It seems that mental retardation and developmental problems in Nager syndrome are secondary to hearing dysfunction, which was not observed in our patient. While sensorineural hearing loss is not generally associated with Nager syndrome, this has not been well documented. Ear deformities have been described in 88% of the patients reported (24). Our patient had external malformations of the ear including low set ears and bilateral canal artesia. Hearing levels should be assessed during early infancy using electrophysiologic testing such as measurement of auditory brainstem responses and later confirmed using behavioral audiometry. 

Speech difficulties can also arise from impaired hearing, as well as from velopharyngeal insufficiency (21). Cleft palate is common in Nager syndrome (3, 6, 18, 21). This problem is associated with the influence of mandibular retrusion in respiration and feeding.

Palate deformities and necessary interventions such as tracheotomy and gastrostomy also impair vocalization and early speech activities (26). Due to very limited jaw opening in some cases and coexisting limb abnormalities, maintenance of adequate oral hygiene may represent a major problem, and self-care may be impossible (2). Although anomalies of the distal upper limb have been reported in 100% of patients with Nager syndrome with a notable inclusion of the thumbs (24), our patient had no thumb deformity and only the upper limbs were problematic. Our patient was female and had no external genital deformities.

In this case, multidisciplinary management by a craniofacial team is needed. Early intervention, intensive education, new surgical techniques, and an emphasis on coordinated care have improved the quality of life for this patient with Nager syndrome. After emergency interventions such as a tracheotomy and gastrostomy for respiratory and feeding problems, correction of some functionally restrictive anomalies is possible. Cooperation between pediatrics, otolaryngology-head and neck surgery, plastic surgery, dentistry, oral surgery, orthopedic surgery, genetics, developmental psychology, developmental pediatrics, audiology and speech/language pathology is needed to optimize the treatment strategy.
